# Screening outcome of HPV-vaccinated women: Data from the Danish Trial23 cohort study

**DOI:** 10.1371/journal.pone.0306044

**Published:** 2024-06-25

**Authors:** Mette Hartmann Nonboe, George Maria Napolitano, Caroline Kann, Berit Andersen, Mary Holten Bennetsen, Sanne Christiansen, Anna Poulsgaard Frandsen, Carsten Rygaard, Rouzbeh Salmani, Jeppe Bennekou Schroll, Elsebeth Lynge

**Affiliations:** 1 Centre for Health Research, Zealand University Hospital, Nykøbing Falster, Nykøbing Falster, Denmark; 2 Department of Public Health, University of Copenhagen, Copenhagen, Denmark; 3 Department of Gynaecology, Rigshospitalet, Copenhagen, Denmark; 4 University Research Clinic for Cancer Screening, Department of Public Health Programmes, Randers Regional Hospital, Randers, Denmark; 5 Department of Pathology, Randers Regional Hospital, Randers, Denmark; 6 Department of Pathology, Sydvestjysk Hospital, Esbjerg, Denmark; 7 Department of Pathology, Aalborg University Hospital, Aalborg, Denmark; 8 Department of Pathology, Zealand University Hospital, Roskilde, Roskilde, Denmark; 9 Department of Gynaecology and Obstetrics, Herlev Gentofte University Hospital, Herlev, Denmark; 10 Center for Evidence-Based Medicine Odense (CEBMO) and Cochrane Denmark, University of Southern Denmark, Odense, Denmark; Istituto Nazionale Tumori IRCCS Fondazione Pascale, ITALY

## Abstract

**Background:**

Danish women—who were HPV-vaccinated as girls—are now reaching an age where they are invited to cervical cancer screening. Because of their expected lower cervical cancer risk, we must reassess our screening strategies. We analyzed Danish HPV-vaccinated women’s outcomes after the first screening test at age 23.

**Methods and findings:**

Our study was embedded in Danish routine cytology-based screening. We conducted an observational study and included women born in 1994, offered the 4-valent HPV vaccine at age 14, and subsequently invited to screening at age 23. Cervical cytology was used for diagnostics and clinical management. Residual material was HPV tested with Cobas® 4800/6800. The most severe histology diagnosis within 795 days of screening was found through linkage with the Danish National Pathology Register. We calculated the number of women undergoing follow-up (repeated testing and/or colposcopy) per detected cervical intraepithelial neoplasia (CIN2+). A total of 6021 women were screened; 92% were HPV-vaccinated; 12% had abnormal cytology; 35% were high-risk HPV-positive, including 0.9% HPV16/18 positive, and 20% had follow-up. In women that were cytology-abnormal and HPV-positive (Cyt+/HPV+), 610 (98.5%) had been followed up, and 138 CIN2+ cases were diagnosed, resulting in 4.4 (95% CI 3.9–5.2) women undergoing follow-up per detected CIN2+. In contrast to recommendations, 182 (12.2%) cytology-normal and HPV-positive (Cyt-/HPV+) women were followed up within 795 days, and 8 CIN2+ cases were found, resulting in 22.8 (95% CI 13.3–59.3) women undergoing follow-up per detected CIN2+.

**Conclusion:**

Overall, HPV prevalence was high in HPV-vaccinated women, but HPV16/18 had largely disappeared. In the large group of cytology-normal and HPV-positive women, 23 had been followed up per detected CIN2+ case. Our data indicated that primary HPV screening of young HPV-vaccinated women would require very effective triage methods to avoid an excessive follow-up burden.

**Trial registration:**

**Trial registration number:** NCT0304955.

## Introduction

Persistent infection with high-risk human papillomavirus (HPV) is the leading cause of cervical cancer [1]. The first HPV vaccine was marketed in 2006, and out of the 12 known carcinogenic HPV types, the vaccine was approved to protect against HPV 16 and 18, which are estimated to account for about 70% of cervical cancers [2]. Randomized controlled trials (RCT) showed that the HPV vaccine protected against severe vaccine-type-related cervical intraepithelial neoplasia (CIN) in women who were HPV-naïve at the time of vaccination [3]. Observational studies of vaccinated compared to non-vaccinated women have further strengthened the evidence of a cancer-protective effect of HPV vaccination [4, 5].

In parallel with vaccine development, HPV assays were tested as alternatives to cytology in cervical screening. In 2014, a meta-analysis found that HPV-based screening provided better protection against cervical cancer than cytology-based screening [6]. Consequently, many screening programs have changed to primary HPV screening for women aged 30 and above [7–12]. Due to frequent transient HPV infections, the balance between the benefits and harms of primary HPV screening may be less favorable in younger women [13]. Nevertheless, several countries, including Australia [14], Norway [15], and England [16], have implemented primary HPV screening from the age of 25 and Sweden from the age of 23 [17].

Historically, women in Denmark had a high background risk of cervical cancer [18]. Population-based cytology screening has been ongoing for decades, and Denmark was one of the first countries to introduce HPV vaccination [19]. We conducted an observational method-study (Trial23) to map the profile of HPV infections and screening outcomes of HPV-vaccinated women [20]. We included women born in 1994, which were offered HPV vaccination at age 14, and invited to a screening at age 23. The study was embedded in the routine screening program, where diagnostics and clinical management are based on cytology. The residual cell material was HPV tested to identify prevalent HPV infection [21] and the results of the first screening is reported in this paper. We combined cytology and HPV test results and determined the number of women undergoing follow-up per detected high-grade lesion.

## Materials and methods

### Cervical cancer prevention in Denmark

From October 2008, all girls aged 13–15 were offered HPV vaccination in Denmark at no cost, and from January 2009, the vaccine was offered to all girls turning 12 years old. The 4-valent HPV vaccine was offered until February 2016, then replaced by the 2-valent vaccine until November 2017, and hereafter replaced by the 9-valent vaccine [22]. Women vaccinated with the 9-valent vaccine will reach screening age in 2029, and this cohort is expected to have an even lower risk of cervical cancer.

Cervical screening is offered to women aged 23–64. Women are personally invited, and general practitioners collect cervical samples. Since January 2021, the Danish screening program has been as follows: Women aged 23–29 are invited to screening every third year, and their cervical samples are analyzed by examining the cell morphology (cytology). Women aged 30–49 and born on an uneven day are screened with HPV every fifth year, and women aged 30–49 born on an even day are screened with cytology every third year. Women aged 50–59 are invited every fifth year for either HPV or cytology test depending on whether they were born on an uneven or even day. Women aged 60–64 are offered an HPV check-out test [23].

### Study population

As reported previously [21, 24], Trial23 was embedded in the routine cytology-based screening program and conducted across three and a half of Denmark’s five regions. The remaining one-and-a-half regions did not participate because the pathology departments did not have sufficient capacity. Trial23 included all women born in 1994, residing in Denmark at the age of 14, and present in the study area at the age of 23. In January 2017, women in this closed cohort were randomly allocated to have cytology screening only or, in addition, to have the residual cell material from their screening test HPV tested. Data collection started on February 1, 2017. We included all cell samples collected after this date regardless of whether they were screening samples, symptom-initiated samples, or samples taken as part of follow-up. In this paper, we only report the results from women who had HPV tests performed.

### Method

General practitioners collected cell samples in SurePath® liquid-based medium (BD, Franklin Lakes, NJ, USA), and the samples were analyzed at the regional pathology departments. Cytology results were coded according to the Bethesda classification and divided into five groups: 1) negative for intraepithelial lesion or malignancy (NILM); 2) atypical squamous cells of undetermined significance (ASCUS); 3) low-grade squamous intraepithelial lesion (LSIL); 4) atypical squamous cells cannot exclude HSIL (ASCH) or atypical glandular cells (AGS); and 5) high grade squamous intraepithelial lesion (HSIL) or adenocarcinoma in situ (AIS) (S1 Table in S1 File).

Residual sample material was HPV tested with Cobas® 4800 HPV-DNA test (Roche Diagnostics, Switzerland) and in one region (Region Zealand) with Cobas® 6800 (Roche Diagnostics, Switzerland) from February 2021 onwards. Given the comparability of Cobas® 4800 and 6800, stratification by technology was unnecessary [25]. Cobas® 4800/6800 has four channels: HPV-16; HPV-18; a batch channel for 12 HPV types (31, 33, 35, 39, 45, 51, 52, 56, 58, 59, 66, and 68) henceforth called ‘other HPV types’; and a β-globin channel serving as control. Cytology should be analyzed without knowing the HPV test, and clinical management should rely only on the cytology results. However, the sequence of tests had to follow the standard laboratory procedures, which varied across laboratories and over time.

As all women with abnormal cytology, including HSIL/AIS, ASCH/AGC, LSIL, or ASCUS, and the small group of women with unsatisfactory cytology are recommended follow-up, we merged the groups and used the terms cytology-positive and Cyt+. For NILM, we used the terms cytology-negative and Cyt-. We used the term HPV+ for HPV-positive test results, and for HPV-negative test results, HPV-.

### Follow-up

Women born in 1994 were included when their first cell sample was taken after February 1, 2017. Follow-up data were retrieved on July 2, 2021, from the Danish National Pathology Register. To ensure a follow-up period of 795 days, we ended up including women with a first cell sample taken between February 1, 2017, and March 28, 2019. We chose 795 days to ensure sufficient follow-up time for abnormal findings [23] and to avoid spillover from the second screening round (three years later). Women who died or emigrated within the 795 days and the small number of women with inconsistent codes were excluded from the analysis.

Follow-up depended on the result of the first cell sample. Women with HSIL/AIS or ASCH/AGC test results were recommended direct referral for colposcopy, always including portio biopsy and endocervical curettage/cell sample [23]. Women with ASCUS/LSIL test results were recommended a new cell sample analyzed as cytology test after six months. We defined a *follow-up* as any extra cytology and/or histology sample within 795 days. Based on the most severe histological diagnosis, we reported the number of women with CIN2+ or CIN3+ diagnosed within 795 days.

### Data

The study population was identified from the Danish Central Person Register on January 1, 2017, and data on deaths and emigrations in the follow-up period were retrieved from the same register on July 2, 2021 (S4 Table and S1 Fig in S1 File). Data on HPV vaccinations were retrieved from the National Health Services Register on July 6, 2017, for vaccines given as part of the free vaccination program. Data and the limited number of self-paid vaccines were retrieved from the Prescription Register on the same date (S2 Table in S1 File). We defined a woman as HPV-vaccinated if she had received at least one dose. Data on HPV test results were retrieved from the pathology departments on January 31, 2021. Data on cytology and histology diagnoses were retrieved from the Danish National Pathology Register on July 2, 2021. Unique personal identification numbers were used for register linkages. All data were pseudonymized and stored at Statistics Denmark.

### Statistical analysis

We reported diagnostic outcomes based on the women’s initial screening test (defined by a combination of the cytology and HPV test results). The primary outcome was the number of women undergoing follow-up per detected CIN2+ case. The secondary outcome was positive predictive value (PPV), defined as the number of detected CIN2+ cases out of all women who had follow-up. Danish data protection legislation prohibits us from reporting numbers less than five; for sums of numbers greater than and less than five, we used “<” (e.g., 8+<5 = <13). 95% confidence intervals (95% CI) for proportions were computed as Wilson intervals with continuity correction and for number of women in follow-up per detected CIN2+ via bootstrap (with 5000 replicates). The average time to follow-up was calculated as the mean of the time difference between the date of the first cell sample and the date of the sample with the most severe diagnosis in the follow-up period of 795 days. In a sub-analysis, HPV test results were stratified by individual HPV vaccination status.

Data handling and statistical analyses were performed at a server in Statistics Denmark using SAS software, Version 9.4. Copyright © 2016 SAS Institute Inc.

### Ethics

The Ethical Committee of the Capital Region of Denmark deemed the study a method-study (H-16022292), for which informed consent was not required. The Danish Data Protection Agency approved the study under the University of Copenhagen (SUND-2016-22). Permission for data extraction was obtained from the Danish Patient Safety Authority (3-3013-2328/1). HPV-DNA test kits for the study were provided free of charge by Roche.

## Results

Between February 1, 2017, and March 28, 2019, cell samples were received from 6,364 women who had both cytology and HPV testing. Following the exclusion of 162 women due to emigration or death and 161 women due to coding errors, the final study population comprised 6021 women. Half the study population was recruited in the Central Region: 3021 (50.2%) women. The remaining women were distributed as follows: 1249 (20.7%) from Region North, 1130 (18.8%) from Region Zealand, and 621 (10.3%) from Region South (Table 1). The women’s ages ranged from 22 to 25 at the time of their first cell sample, with the majority (4766; 79.2%) being 23. Nearly all, 5548 (92.1%), were HPV-vaccinated with at least one dose. Cytology results from the first cell sample showed that 5300 (88.0%) of the women had negative cytology, 671 (11.2%) had positive cytology, and 50 (0.8%) had unsatisfactory cytology. The HPV test was negative for 3916 (65.0%) of the women, while 2105 (35.0%) women had a positive HPV test. Only 42 women had an HPV16-positive test, and nine had an HPV18-positive test; in total, 51 (0.9%) women had an HPV 16/18 positive test.

**Table 1:** Characteristics of the study population at the first cell sample. The cohort consist of Danish women born in 1994. They were all offered free HPV vaccination at age 14 and invited to cytology screening at age 23.

Number of women	6021 (100%)	Number of women	6021 (100%)
Region		First cytology result	
• North	1249 (20.7%)	- Unsatisfactory	50 (0.8%)
• Central	3021 (50.2%)	- NILM	5300 (88.0%)
• South	621 (10.3%)	- ASCUS	342 (5.7%)
• Zealand	1130 (18.8%)	- LSIL	262 (4.4%)
Age at first sample		- ASCH/AGC	24 (0.4%)
• 23 years	4766 (79.2%)	- HSIL/AIS	43 (0.7%)
• 24 years	814 (13.5%)	HPV test result	
• 25 years	50 (0.8%)	- Negative	3916 (65.0%)
HPV-vaccinated		- Positive	2105 (35.0%)
• Yes^a^	5548 (92.1%)	- HPV 16/18^b^	51 (0.9%)
• No	473 (7.9%)	- Other HPV only^c^	2054 (34.1%)

^a^A least one dose

^b^Including possible co-infections with other high-risk HPV types

^c^HPV types 31, 33, 35, 39, 45, 51, 52, 56, 58, 59, 66, and 68

Abbreviations

NILM = negative for intraepithelial lesion or malignancy, ASCUS = atypical squamous cells of undetermined significance, LSIL = low grade squamous intraepithelial lesion, ASCH = atypical squamous cells cannot exclude HSIL, AGC = atypical glandular cells, HSIL = high grade squamous intraepithelial lesion, AIS = adenocarcinoma in situ

Among the 6021 women included, 721 (12.0%) women were cytology-positive, 686 (95.1%) of these women had been followed up within 795 days, and 143 (2.4%) were diagnosed with CIN2+. The numbers correspond to a PPV for CIN2+ of 20.8%, where 4.8 (95% CI 4.2–5.7) women had been followed up per detected CIN2+ case (Table 2). Of the 5300 women with negative cytology, 495 (9.3%) women had been followed up, and <13 (<0.2%) were diagnosed with CIN2+.

**Table 2:** Follow-up and detected CIN2+ and CIN3+ by a combination of cytology and HPV test results at the first cell sample.

	Total	Cyt+			Cyt-			Total
	women	Total	HPV+	HPV-	Total	HPV+	HPV-	HPV+
**Screened women**	6021	721	619	102	5300	1486	3814	2105
**(% by row)**	-100%	-12.00%	-10.30%	-1.70%	-88.00%	-24.70%	-63.30%	-35.00%
**- Of which HPV 16/18**	51	20	20	0	31	31	0	51
**Follow-up**	1181	686	610	76	495	182	313	792
**(% by column)**	-19.60%	-95.10%	-98.50%	-74.50%	-9.30%	-12.20%	-8.20%	-37.60%
**- Of which HPV 16/18**	29	19	19	0	10	10	0	29
**Average days to follow-up**	353	282	284	270	452	451	453	322
**(SD)**	-239	-213	-214	-201	-237	-241	-236	-231
**CIN2+ detected**	151+<5	143	138	5	8+<5	8	<5	146
**(% of total screened women)**	(<2.6%)	-2.40%	-2.30%	-0.10%	(<0.2%)	-0.10%	(<0.1%)	NR
**- Of which HPV 16/18**	10	<5	<5	0	<5	<5	0	10
**PPV in %: CIN2+/Follow-up**	<13.2%	20.80%	22.6%^a^	6.6%^a^	<2.6%	4.40%	<1.2%	NR
**Follow-up/CIN2+**	>7.6	4.8	4.4	15.2	>35.8	22.8	>90.6	NR
**(95% CI)**	(6.7–9.1)	(4.2–5.7)	(3.9–5.2)	(7.9–65.0)	(30.0–117.4)	(13.3–59.3)	(58.6–334.0)	NR
**CIN3+ detected**	75+<5	72	≥70	<5	<5	<5	0	75
**(% of total screened women)**	(<1.3%)	-1.20%	(≥1.2%)	(<0.1%)	(<0.1%)	(<0.1%)	0	NR
**PPV in %: CIN3+/Follow-up**	<6.8%	10.50%	≥11.5%	<6.6%	<1.0%	<2.7%	-	NR
**Follow-up/CIN3+**	>14.8	9.5	<8.7	>15.2	>99.0	>36.4	-	NR
**(95% CI)**	(12.8–19.8)	(7.8–12.1)	(7.0–11.0)	(20.3–91.0)	(60.0–498.0)	(22.0–184.0)	-	NR

^a^ The PPVs of the Cyt+/HPV+ and Cyt+/HPV- groups were found to be statistically significantly different: p-value (Chi-squared test) = 0.001.

NR: Not relevant because HPV+ was not followed up per se. Number <5 and possible recalculation not reported.

Abbreviations: Cyt+ cytology-positive; Cyt- cytology-negative; HPV+ HPV-positive; HPV- HPV negative; CIN cervical intraepithelial neoplasia; PPV positive predictive value; SD Standard deviation; CI confidence interval

When dividing the results based on both initial cytology and HPV test, we found that 619 (10.3%) women had a Cyt+/HPV+ test result, 102 (1.7%) had a Cyt+/HPV- result, 1486 (24.7%) had a Cyt-/HPV+ result, and 3814 (63.3%) had a Cyt-/HPV- result.

Among the 619 women with a Cyt+/HPV+ test result, 610 (98.5%) women had a follow-up, and 138 (2.3%) were diagnosed with CIN2+, corresponding to a PPV for CIN2+ of 22.6%, with 4.4 (95% CI 3.9–5.2) women followed up per detected CIN2+ case. A smaller group of 102 (1.7%) women had a Cyt+/HPV- test result, of whom 76 (74.5%) had a follow-up, and five women were diagnosed with CIN2+, resulting in a PPV for CIN2+ of 6.6%, with 15.2 (95% CI 7.9–65.0) women followed up per detected CIN2+ case. A large group of 1486 (24.7%) women had a Cyt-/HPV+ test result; 182 (12.2%) had a follow-up, and eight were diagnosed with CIN2+. The numbers resulted in a PPV for CIN2+ of 4.4%, with 22.8 (95% CI 13.3–59.3) women followed up per detected CIN2+ case. In the largest group of 3814 (63.3%) women with Cyt-/HPV- test results, surprisingly, 313 (8.2%) women had been followed up, and <5 women were diagnosed with CIN2+. Based on small numbers and compared with the CIN2+ data, approximately double the number of women had been followed up per detected CIN3+ case.

Approximately half of the women with follow-up had undergone colposcopy with biopsies, and the other half had undergone repeated cell sampling only. The average time to follow-up was 284 (214 standard deviation (SD)) days for Cyt+/HPV+, 270 (SD 201) days for Cyt+/HPV-, 451 (SD 241) days for Cyt-/HPV+, and the same 453 (SD 236) days for Cyt-/HPV-. Ninety-two percent of women in the cohort had been HPV-vaccinated, and only 51 (0.9%) were HPV 16/18 positive at the time of their first cell sample. For HPV-vaccinated women, this number was 20 (0.4%), and for non-vaccinated women, it was 31 (6.6%) (S3 Table in S1 File).

## Discussion

### Main finding

We studied a cohort of women, of whom 92% were HPV-vaccinated with the 4-valent vaccine at age 14. Women were recruited at their first cervical cell sample after turning 23 and followed up for 795 days after recruitment. The recommended clinical management was based on the cytology outcome. Twelve percent had positive cytology; 11% had been followed up; 2.4% had CIN2+; 1.2% had CIN3+; and 4.8 women had been followed up per detected CIN2+ case.

Thirty-five percent of the samples were HPV-positive, almost exclusively with HPV types other than 16/18. Positive HPV tests were not recommended for follow-up per se. Nevertheless, 20% of the cohort members had been followed up. For women with negative cytology, follow-up time was independent of the HPV test result, indicating that the clinical management was not affected by the HPV test result. For all cytology-positive women, 4.8 women had been followed up per detected CIN2+ case. For Cyt+/HPV+, 4.4 women had been followed up per detected CIN2+ case. In the small group of Cyt+/HPV- women, this number reached 15.2, indicating the importance of HPV triage in cytology-positive women. For the large group of women with Cyt-/HPV+, 22.8 women had been followed up per detected CIN2+ case. These numbers doubled per detected CIN3+ case. In women with negative cytology and positive HPV test, detection of CIN2+ and CIN3+ thus came at a considerably more significant burden of follow-up than in women with positive cytology.

### Strengths and limitations

It was a strength that our project was embedded in the routine screening program in Denmark, and our results reflected procedures in this program and similar programs. The random allocation of women for HPV testing ensured that the collected HPV data represented the studied birth cohort. Linking data from pathology and population registers ensured complete follow-up and vaccination status data. With a study population of about 6000 women and a vaccination coverage of 92%, we were not able to distinguish between vaccination effects and herd immunity.

It’s important to note the limitations of our study. As the study was embedded in the routine cytology-based screening program, it should be stressed that our results cannot be interpreted as showing the effects of primary HPV screening.

Cytology was intended to be read independently of the HPV result, but we had to follow the standard procedure of the respective laboratories. There was probably some spillover from the outcome of the HPV test results on cytology reading. A tendency to upgrade cytology in the presence of awareness of a positive HPV test has been reported earlier [26–28]. There was possibly also some spillover from the HPV test results on follow-up of cytology-positive women. Among Cyt+/HPV+ women, 98.5% had been followed up. In comparison, 74.5% of Cyt+/HPV- women had been followed up. This imbalance could affect the comparison between Cyt+/HPV+ and Cyt+/HPV- women but would only marginally affect the more important comparison between all Cyt+ and Cyt-/HPV+ women.

As a follow-up of Cyt-/HPV+ women was not recommended, we cannot exclude a selection bias amongst those followed up, for instance, the occurrence of symptoms as irregular bleeding. Therefore, any bias in the follow-up of Cyt-/HPV+ women is expected to result in fewer women followed up per detected CIN2+ than we would have seen if all Cyt-/HPV+ women had been followed up. The possible bias will, therefore, result in a conservative estimate of the number of women followed up per detected CIN2+ case. Finally, number of detected CIN3+ cases was small, and possible misclassification of CIN2 cases could not be excluded.

### Interpretation

In Australia, HPV screening of women offered the HPV vaccination at a young age (women <25) showed a similar proportion of HPV-positive women (~32% vs. 35%) and a similar proportion of HPV16/18 positive (1.5% vs. 0.9%) [9]. The HPV vaccination initiative in Australia commenced in 2007 for women aged 12–26 [29], with primary HPV screening implemented on December 1, 2017, targeting women aged 25–74. The Australian data were reported for women screened between December 1, 2017, and December 31, 2019, and also included data on women <25 [9]. As our dataset lacked a distinction between screening and non-screening samples, we included all Australian samples for comparison (Tables A4 and A6 in reference 9). Despite Denmark’s higher vaccination coverage, the proportion of HPV-positive women in the Australian data was reasonably similar to what we found.

The Australian CIN2+ data were reported only for women with screening samples (Tables A4, A8, A10, and A17 in reference 9). Among women testing positive for HPV 16/18, four had a histology follow-up per detected CIN2+. In women who tested positive for HPV types not 16/18 with high-grade cytology, two had a histology follow-up per detected CIN2+. In women who tested positive for HPV types not 16/18 with NILM/low-grade cytology and with a repeated test of HPV-positive with high-grade cytology, 2.1 women had a histology follow-up per detected CIN2+. Finally, in the largest group of women positive for HPV types not 16/18 with NILM/low grade cytology, 9.4 had a histology follow-up per detected CIN2+. Despite the different cut-off values used in the Australian and Danish data, both datasets pointed to a considerable follow-up burden for detecting CIN2+ in young women positive for HPV not 16/18 and where all (in Denmark) and the majority (in Australia) were cytology normal.

Observational data from the English pilot program, initiated in 2013, compared primary HPV screening with cytology screening in young women aged 24–29 [30]. When comparing one round of primary HPV screening with two rounds of cytology screening for women aged 25 at first screen, the English pilot project showed a 43% reduction in the number of screens, with a 109% increase in positive screening tests; colposcopy referral increased by 34%; and detected CIN2/3 increased by 18%. The authors concluded that the “balance between the clinical benefits and the harms of [high-risk]-HPV testing in women younger than 30 is more favorable than has been considered so far” [30]. Since 2015, HPV-vaccinated entered the pilot program, the prevalence of HPV 16/18 decreased from 13% to 3%, while the prevalence of other high-risk HPV types oscillated between 25% and 27% [31]. Although direct comparison with Danish data may be limited due to differences in pre-vaccination HPV prevalence (34% in English women aged 24–25 [31] versus 45% in Danish women aged 20–29 [24]), the substantial reduction in HPV 16/18 prevalence, combined with the stable prevalence of other HPV types, suggests a similar trend.

The 1994-cohort in our study was offered HPV vaccination at the age of 14. While most women were likely HPV-naïve at the time of vaccination, approximately 24% of girls in this cohort reported being sexually active at that age [32]. From 2009, HPV vaccination was offered from age 12, potentially resulting in even lower HPV16/18 prevalence in subsequent cohorts. However, temporary drops in vaccination coverage may have disrupted this pattern [33].

Since November 2017, the 9-valent HPV vaccine has been used in the Danish vaccination program, protecting against HPV 16, 18, 31, 33, 45, 52, and 58 [34]. These latter types constitute over half of the remaining high-risk HPV prevalence after eliminating HPV 16 and 18. [35]. The first cohort vaccinated with the 9-valent HPV vaccine will reach screening age in 2029, marking a considerable reduction in cervical cancer risk among young Danish women compared to those born in 1994. Future screening recommendations for young women should be seen in this perspective, focusing on effective management of HPV types other than 16/18 to ensure that screening benefits outweigh potential harms. We have shown that being HPV-positive with types not 16/18 requires the availability of effective triage methods.

## Conclusion

HPV prevalence for HPV type not 16/18 was high among HPV-vaccinated women. One-fourth of the women in our cohort were cytology-negative and HPV-positive and follow-up of many women was required to detect one CIN2+ case. Our data indicated that primary HPV screening with follow-up of all positive test results in vaccinated women would imply a considerable burden of repeated testing and/or colposcopy unless a very effective triage was implemented.

## Supporting information

S1 File (DOCX)

## Abbreviations

AGS: Atypical glandular cells
AIS: Adenocarcinoma in situ
ASCH: Atypical squamous cells cannot exclude HSIL
ASCUS: Atypical squamous cells of undetermined significance
CI: Confidence interval
CIN: Cervical intraepithelial neoplasia
Cyt+: Cytology-positive/abnormal
Cyt-: Cytology normal
HPV: High-risk human papillomavirus
HPV+: HPV-positive
HPV-: HPV negative
HSIL: high grade squamous intraepithelial lesion
LSIL: Low-grade squamous intraepithelial lesion
NILM: Negative for intraepithelial lesion or malignancy
PPV: Positive predictive value
RCT: Randomized controlled tria
SD: Standard deviation
