# Supplementary material for: Screening outcome of HPV-vaccinated women: Data from the Danish Trial23 cohort study
Source: PLoS One. 2024 Jun 25;19(6):e0306044. doi: 10.1371/journal.pone.0306044 (PMC11198772; doi:10.1371/journal.pone.0306044)
Supplement: S1 File — (DOCX) [file pone.0306044.s001.docx]

Zealand University Hospital, Nyk. F. January 9 2024, revised May 21 2024

**Nonboe et al. Screening outcome of HPV-vaccinated women: data from the Danish Trial23 cohort study**

**Supporting information**

S1 Table. SNOMED (Systemized Nomenclature of Medicine) codes for cytology and histology

| **Cytology codes (T-codes T8X2**, T8X3**)** | |
| --- | --- |
| **Diagnose** | **M-Codes** |
| Unsatisfactory | M09000 M09010 M09011 M09012 M09013 M09014 M09015 M09016 M09017 M09018 M09019 M0901X M0901Y M09070 M09100 M09140 M09145 M09150 M30610 M37000 M54310 |
| NILM | M00100 M00120 M00121 M00122 M01111 M02561 M09450 M09460 M09462 M09463 M11600 M11610 M51620 M69520 M69784 M69810 M69820 M69880 M74030  M69780  MYY122 (excluding M09010)  M4**** (excluding M09010)  M58***  M72***  M73*** (excluding M73005 M73225 M73229 M73309) |
| ASCUS | M67014 M67020 M69700 M69711 M69712 M69762 M72125 M73005 M73225 |
| LSIL | M67016 M69701 M76700 M76701 M76720  M69790 (excluding M67014 M69711 M72125 M73005 M733225 M69700)  M74A*9 |
| ASCH | M67014 M69711 M72125 M73005  M73225 M69700 |
| AGC | M67020 M69712 M69762 |
| HSIL | M67017 M69702 M69703 M69760 M73229 M74HG9 M80102 M80702 M80722 M80732 M80762 M80812  M740.9  M74C.9  M74B.9 |
| AIS | M81402 M73309 |
|  | |
| **SNOMED table - Histology codes (T-codes T83***, T820**, T829**)** | |
| **Diagnose** | **M-Codes** |
| Unsatisfactory | M09000 M09010 M09011 M09013 M09014 M0901U M09070 M09100 M09145 M09150 |
| Normal | M00100 M00120 M01111 M09450 M09451 M09462 M28000 M31050 M33400 M51620 M54000 M72120 M73220 M74030 M76500 M76800 M79500 M79810 M88100 M88900 M90840  M116**  M4****  M58***  M72***  M73*** |
| CIN1 | M69790 M76700 M76701 M76720  M74A*9 |
| CIN2 | M74B*9  M740*9 |
| CIN3 | M74C*9  M8***2 |
| Cancer | M8***3  M9***3 |

S2 Table. Overview of codes used from Danish National health registers

| **Codes from the National Register of Pathology (NRP) for cervical screening outcomes^a^** | |
| --- | --- |
| Cervical histology | T83* (cervix uteri)  T820* (uterus)  T829* (corpus and cervix uteri) |
| Cervical cytology | T8X3* (cervical cytology) |
| **Codes from the Danish National Prescription Register for HPV vaccination^b^** | |
| Quadrivalent HPV vaccine | J07BM01 |
| Bivalent HPV vaccine | J07BM02 |
| **Codes from the National Health Services Register (NHSR) for HPV vaccination^c^** | |
| HPV vaccine 1^st^ dose | 808328 |
| HPV vaccine 2^nd^ dose | 808329 |
| HPV vaccine 3^rd^ dose | 808330 |

S2 Table legend:

All registers in Denmark use unique personal identification numbers (CPR numbers) as a variable.

^a^The register includes detailed information on all pathology specimens analysed in Denmark**.**

^b^The register includes individual-level data on dispensed prescriptions.

^c^The register includes information on all primary health care contacts. In Denmark, childhood vaccination is undertaken by general practitioners

S3 Table. HPV test result in Danish women born in 1994 by vaccination status

|  | **Total** |
| --- | --- |
| **All screened women** | 6021 |
| - **HPV Negative** | 3916 |
| - **HPV Positive** | 2105 |
| - 16/18 | 51 |
| - Other only^a^ | 2054 |
| **HPV vaccinated women** | 5548 |
| - **HPV Negative** | 3607 |
| - **HPV Positive** | 1941 |
| - 16/18 | 20 |
| - Other only^a^ | 1921 |
| **Non HPV vaccinated women** | 473 |
| - **HPV Negative** | 309 |
| - **HPV Positive** | 164 |
| - 16/18 | 31 |
| - Other only^a^ | 133 |

S3 Table legend:

^a^HPV types 31, 33, 35, 39, 45, 51, 52, 56, 58, 59, 66, and 68

S4 Table. Data sources

| **Register** | **Data of data retrieval** | **Retrieval criteria** | **Varibles retrieved for each woman fulfilling the retrieval criteria** |
| --- | --- | --- | --- |
| **Danish Central Person Register** | January 1, 2017 | Women born 1994, living in Denmark at age 14, living in study area on 1 January 2017 | Personal identification number (PIN) |
| **Danish Central Population Register** | July 2, 2021 | As above | Dates of death and emigration |
| **National Health Services Register and Prescription Register** | July 6, 2017 | As above | HPV1 (= having received at least one dose of HPV) and date of first vaccination |
| **Pathology departments** | January 31, 2021 | As above | For each HPV-test: HPV16, HPV18, other high risk HPV-types and date of sample |
| **Danish National Pathology Register** | July 2, 2021 | As above | Cytology and histology diagnoses and date of sample |



S1 Fig. Time scale for events
